# Relationship of Serum Total Insulin-Like Growth Factor Binding Protein-3 with Insulin-Like Growth Factor-I and Glucose Tolerance in Korean Children and Adolescents

**DOI:** 10.1155/2021/9966114

**Published:** 2021-06-22

**Authors:** Sun-Young Kim, Minsun Kim, Youngman Oh, Dae-Yeol Lee

**Affiliations:** ^1^Research Institute of Clinical Medicine of Jeonbuk National University-Biomedical Research Institute of Jeonbuk National University Hospital, Jeonju 54907, Republic of Korea; ^2^Department of Pediatrics, Jeonbuk National University Medical School, Jeonju 54907, Republic of Korea; ^3^Department of Pathology, School of Medicine Medical College of Virginia Campus, Virginia Commonwealth University, Richmond, VA 23298, USA

## Abstract

Insulin is important in glucose metabolism. However, insulin-like growth factor binding protein (IGFBP) also plays an important role in glucose homeostasis, although the IGF-independent role of IGFBP-3 in the glucose intolerance state is poorly understood. We investigated the relationship of serum IGF-I with total IGFBP-3 levels and glucose tolerance in Korean children and adolescents who underwent the oral glucose tolerance test (OGTT). A total of 187 children without known diabetes underwent OGTT, and data related to their clinical and laboratory parameters were collected. Serum IGF-I and total IGFBP-3 levels, fasting plasma glucose levels, lipid profiles, insulin levels, C-peptide levels, homeostasis model assessment of insulin resistance (HOMA-IR) index, and glycated hemoglobin (HbA1c) levels were measured. Serum IGF-I and total IGFBP-3 levels were significantly higher in individuals with impaired glucose tolerance and type 2 diabetes (DM) than in those with normal glucose tolerance (NGT) (*P* < 0.05). Serum IGF-I and IGFBP-3 levels were correlated with age, HbA1c, C-peptide, insulin, and HOMA-IR in the NGT group. However, these relationships were altered in patients with glucose intolerance, especially in those with DM. In the DM group, serum IGF-I and total IGFBP-3 levels were positively correlated with fasting plasma glucose and HbA1c levels. In addition, total IGFBP-3 levels were positively correlated with total cholesterol and low-density lipoprotein cholesterol and IGF-I levels but not with age or body mass index. The IGF-I-IGFBP-3 axis, especially IGFBP-3, may be involved in the pathogenesis and metabolic control of glucose intolerance, specifically in diabetes patients. Moreover, IGFBP-3 might be a therapeutic marker.

## 1. Introduction

Type 1 diabetes is the most common hyperglycemic disease in children and adolescents, but the incidence of type 2 diabetes has significantly increased due to the recent increase in childhood obesity [1]. Recently, in Korea, the frequency of type 2 diabetes and prediabetes has also increased in teenagers and young adults [2]. Thus, it is important to determine the factors that affect their diagnosis and treatment, control these factors, and find factors that can be used as treatment targets in children and adolescents.

The insulin-like growth factor (IGF) system is important in the regulation of growth and cellular proliferation in the human body [3, 4]. The IGF system includes IGF-I/II peptides, IGF-I and IGF-II receptors, and IGF-binding proteins (IGFBPs) [5–7]. IGFBPs are classified into two groups: a binding protein (IGFBP-1 to -6) that has a high affinity for IGF and an IGFBP-related protein (IGFBP-rP1-10 and IGFBP-rP1, also known as IGFBP-7) that has a low affinity for IGF [8]. IGFBPs transport IGFs to their receptors [8, 9]. The pivotal IGFBP species in the serum is IGFBP-3; it binds to 90% or more of circulating IGF-I and makes a large ternary complex with acid-labile subunits and IGFs [8, 10]. Furthermore, IGFBP-3 has shown cell growth inhibition and apoptosis by IGF-independent activity in various cell types [11]. It has been reported that the IGF-I/IGFBP-3 ratio can be used as an important indicator for the effect of growth hormone treatment in children [12], but there are limited studies conducted in conditions such as metabolic abnormalities and diabetes.

IGF-I may be involved in maintaining glucose homeostasis and lipid metabolism. IGF-I increases insulin sensitivity, peripheral glucose levels, and fatty acid uptake and decreases hepatic glucose production [13]. In addition, positive correlations have been reported between insulin resistance and increased IGF-I levels among patients with diabetes [14, 15]. A study reported that low and high IGF-I levels were both related to a greater risk of type 2 diabetes (DM) [16]. Rajpathak et al. reported a positive association between IGFBP-3 and the risk of DM in women [17]. However, the precise mechanisms of action of IGF-I and IGFBP-3 in DM remain elusive.

In this study, we investigated the relationships of serum IGF-I with total IGFBP-3 levels and glucose tolerance in children and adolescents.

## 2. Materials and Methods

In total, 191 children and adolescents who visited Jeonbuk National University Children's Hospital to diagnose DM by the oral glucose tolerance test (OGTT) during 2010–2018 were initially included. Subjects were excluded from this study if they had been previously diagnosed with diabetes or had diabetic symptoms (such as polyuria, polydipsia, hyperphagia, and weight loss), ketogenesis, or other chronic diseases. Furthermore, three subjects with positive results for the diabetes-related autoantibodies test such as glutamic acid decarboxylase, islet cell, and insulin antibodies and one subject with the fasting serum C-peptide level under 0.6 ng/mL were also excluded. Finally, 187 children and adolescents (aged 10.08–17.60 years) were enrolled; of these, 130 visited the hospital to undergo glucosuria examination based on school urinary glucose screening [18–21], while the other 57 were referred for obesity checkup. A large-scale school urine screening program was implemented in the Jeonbuk Province area. Glucose oxidase tape (UriScan, YD Diagnostic Corporation, Yongin, South Korea) was used to examine the morning urine samples for glycosuria [18]. If the first urine test result was positive, a second urine test was performed within two weeks. If the second test result was positive, students were recommended to visit the hospital for further evaluation. This study followed a retrospective design. According to the OGTT results, subjects were divided into the normal glucose tolerance (NGT) group and the glucose intolerance group; the latter comprised subjects with impaired glucose tolerance (IGT) and DM.

Subjects' medical history and physical examination were based on their first visit to the hospital. Anthropometrics and body mass index (BMI) were measured by two trained pediatric endocrinologists and compared with the data in the 2017 Korean National Growth Charts [22]. Height (cm) was measured with a Harpenden Stadiometer (Holtain Ltd., Crymych, Wales, UK), and weight (kg) was measured with a digital scale (150 A; Cas Co., Ltd., Seoul, Korea). Height and weight were measured to the nearest 0.1 cm and 0.1 kg, respectively. BMI was calculated as weight in kilograms divided by height in meters squared (kg/m^2^). Overweight was defined as 85th ≤ BMI < 95th by age- and sex-specific percentile, while obesity was defined as BMI ≥ 95th percentile. OGTT (1.75 g/kg, maximum 75 g of glucose) was conducted in the morning after fasting for 12 hours. The diagnostic criteria for DM were 2-hour postload glucose (2 hr PLG) level ≥ 200 mg/dL or fasting plasma glucose (FPG) level ≥126 mg/dL; IGT was defined as 2 hr PLG level of 140–199 mg/dL and NGT as 2 hr PLG level <140 mg/dL according to the World Health Organization criteria based on primary OGTT findings [23]. All the patients had negative test results for diabetes-related autoantibodies, with no evidence of ketogenesis, and a fasting serum C-peptide level above 0.6 ng/mL [24]. Serum IGF-I and total IGFBP-3 levels were measured using enzyme-labeled chemiluminescence immunoassay (IGF-I, DiaSorin SPA, Italy; IGFBP-3, Siemens Medical Solutions Diagnostics, USA).

In addition, FPG, total cholesterol, low-density lipoprotein (LDL) cholesterol, high-density lipoprotein (HDL) cholesterol, triglyceride, insulin, C-peptide, and glycated hemoglobin (HbA1c) levels were measured. Insulin sensitivity was measured using the homeostasis model assessment of insulin resistance (HOMA-IR) index [25].

The study was approved by the Institutional Review Board (IRB) of Jeonbuk National University Research Council (IRB no. 2019-09-028); the need for informed consent was waived by the IRB.

### 2.1. Statistical Analysis

All variables are expressed as mean ± standard deviation (SD). One-way analysis of variance and Tukey's test were performed to evaluate OGTT and BMI in all groups using the SPSS software (version 19.0, SPPS, Chicago, IL, USA). The relationships between IGF-I/IGFBP-3 with clinical and laboratory variables were estimated using Pearson correlation coefficients. Statistical significance was set at a *P* value of < 0.05.

## 3. Results

The demographic features of a total of 187 school children and adolescents are given in Table 1. Among the 187 subjects, 33 were diagnosed with IGT and 39 were diagnosed with DM. The DM group subjects were followed up for over a year, and their fasting serum C-peptide levels remained at over 1.0 ng/mL. The mean age and BMI of all subjects were 12.51 ± 3.45 years and 24.50 ± 5.12 kg/m^2^, respectively, and the BMI was similar between the NGT and glucose intolerance groups (IGT and DM). However, both the IGT and DM groups had female predominance compared to the NGT group (*P*=0.004). Those with a family history of DM among the first-degree relatives were significantly more than those in the NGT group; however, they had no family history of type 1 diabetes.  Table 2 provides the clinical and laboratory characteristics of the study subjects. As expected, both the IGT and DM groups had significantly higher systolic blood pressure and FPG, C-peptide, insulin, HOMA-IR, total cholesterol, LDL cholesterol, triglyceride, and lower HDL cholesterol levels than the NGT group (*P* < 0.05). In addition, serum IGF-I and total IGFBP-3 levels were significantly higher in the IGT and DM groups than in the NGT group (*P* < 0.01), with the highest levels found in subjects with newly diagnosed DM. However, there was no difference in the IGF-I/IGFBP-3 ratio among the three groups.

Because serum IGF-I and total IGFBP-3 showed correlation with BMI, we examined whether serum IGF-I and total IGFBP-3 levels vary with BMI in subjects with glucose intolerance (Table 3). The normal weight group, overweight group, and obesity group included 23 (31.9%), 12 (16.7%), and 37 (51.4%) subjects, respectively. Although there were significant differences in age; systolic blood pressure; and serum C-peptide, insulin, HDL cholesterol, and triglyceride levels between normal, overweight, or obese subjects, serum IGF-I and total IGFBP-3 levels were not different among the three groups (*P* > 0.05). These data suggest that alterations in IGF-I and total IGFBP-3 levels resulted from glucose intolerance, but not from obesity.

Finally, the correlation between IGF-I and total IGFBP-3 levels based on clinical and laboratory parameters of the study subjects was analyzed using the Pearson correlation coefficient (Tables 4 and 5). In the NGT group, serum IGF-I levels were positively correlated with age, FPG, HOMA-IR, HbA1c, serum C-peptide, insulin, and total IGFBP-3 levels, while serum total IGFBP-3 levels were correlated with age, BMI, HOMA-IR, serum C-peptide, insulin, HbA1c, triglyceride, and IGF-I levels. However, these relationship patterns were different in patients with glucose intolerance, especially in those with DM. In DM subjects, serum IGF-I was positively correlated with FPG, HbA1c, and total IGFBP-3 levels, while the serum total IGFBP-3 level was correlated with FPG, HbA1c, and LDL cholesterol levels. In contrast, the serum total IGFBP-3 level was not correlated with age, BMI, serum insulin, or HOMA-IR.

## 4. Discussion

We found that high serum IGF-I and total IGFBP-3 levels were associated with glucose intolerance and that these associations with clinical variables were altered according to glucose tolerance status, suggesting that the IGF-I-IGFBP-3 axis plays an important role in the pathogenesis and metabolic control of glucose intolerance, especially in DM. To the best of our knowledge, this is the first study to report that IGFBP-3 is related to glucose intolerance in children and adolescents with naïve type 2 diabetes.

Insulin is the major regulator of glucose homeostasis. Like insulin, the GH-IGF-IGFBP axis may also play a role in maintaining glucose metabolism. IGF-I is a hormone that resembles insulin in structure and is synthesized mainly by the liver when stimulated by growth hormones. The receptors for IGF-I and insulin are similar [3]; thus, they share several identical signaling pathways and cell and biological responses [3,  26]. Therefore, the distinction between the roles of insulin and IGF-1 as causes of diabetes and progression of insulin-resistant states is unclear. Many experimental and clinical studies have suggested that circulating IGF-I is important for glucose regulation. Several studies have reported a positive correlation between IGF-I, insulin resistance, and glucose metabolism [14, 15]; IGF-I was found to improve blood sugar control and insulin sensitivity in healthy adults and DM patients [13, 27].

IGFBP-3, the most abundant circulating IGFBP, may play an important role in blood glucose control and vice versa with IGF-1 in metabolic effects [28]. IGFBP-3 inhibits the bioactivity of IGF-I through binding, thereby reducing the concentration of free IGF-I in circulation and increasing the risk of DM [27–29]. IGFBP-3 also reduces glucose uptake by insulin through decreased insulin-stimulated glucose transporter-4 translocation to the plasma membrane and threonine phosphorylation of Akt [3]. These findings may suggest that serum IGF-I and IGFBPs levels have an impact on the risk of developing DM.

Some research confirmed the role of the IGF-I-IGFBP-3 axis in normal glucose homeostasis and its possible contribution to the etiopathogenesis of DM [30,  31]. Several studies have shown elevated free IGF-I and IGFBP-3 levels and decreased IGFBP-1 levels in IGT and DM patients [31, 32]. In the current study, serum IGF-I levels were significantly higher in individuals with glucose intolerance and were correlated with FPG and HbA1c, but not with serum C-peptide, insulin, and HOMA-IR. We believe the increased IGF-I level in the glucose intolerance state possibly triggered the pathological condition. Further, the onset of DM and IGT is characterized by insulin resistance, hyperinsulinemia, insulin stimulation of IGF-I production in the liver through the upregulated GH signaling pathway (e.g., upregulated expression of the hepatic GH receptor), and decreased IGFBP-1 level [3,  33]. Hyperinsulinemia, through an increased intraportal insulin concentration, causes an increase in hepatic IGF-I production [34], as seen in this study's results. Accordingly, the high serum IGF-I level in DM patients may be caused by high insulin levels, rather than reflecting the biological role of the IGF axis on DM pathogenesis [35]. In addition, Sandhu et al. [35] found a negative association between IGF-I levels and the risk of developing IGT/DM in patients after 4.5 years of follow-up. Yuen et al. [36,  37] found that serum GH concentration and its direct effects on insulin suppression of hepatic gluconeogenesis are reduced by IGF-I; IGF-I also indirectly enhances hepatic insulin action by increasing fatty acid uptake in muscles. Further, Ricotti et al. [38] reported the relationship between decreased serum IGF-I and high blood glucose level, insulin, and insulin resistance. In a murine model, IGF-I may play an important role in improving glucose tolerance and insulin sensitivity [39]. These data support that an increased IGF-I could inhibit the progression of glucose intolerance states and their complications. Although serum IGF-I is negatively related to HbA1c in DM [40], it was positively correlated with FPG and HbA1c levels in the current study, suggesting a significant negative role for serum IGF-I in metabolic control of DM. Abbas et al. [41] suggested that the absence of prospective studies on the progression of insulin resistance and the unclear measurement of free or total IGF-I concentration caused the various results obtained regarding the relationship of IGF-1/glucose metabolism. Moreover, they reported that the role of IGF-I in glucose homeostasis has not yet been completely elucidated. Therefore, it is necessary to study factors affecting IGF-I bioactivity, such as IGFBPs; further studies are also needed to understand the unclear mechanism.

Recent studies indicated that dysregulated IGF-1/IGFBP levels could cause IGT [42]. Of note, IGFBPs have an important role in the development of obesity, insulin resistance, and metabolic syndrome. Cross-sectional studies revealed that serum IGFBP-3 levels were elevated in patients with DM and positive correlations were observed between fasting insulin and C-peptide levels [26, 43]. In our study, total serum IGFBP-3 levels were significantly elevated in DM patients and were positively correlated with FPG, HbA1c, total cholesterol, and IGF-I levels. IGFBP-3, a slower regulator of free IGF-I than IGFBP-1, is regulated by GH and IGF-I and has marked contrasting and independent physiological effects on peripheral insulin action [28]. Therefore, high IGFBP-3 levels may be a risk factor for DM. Rajpathak et al. reported that serum IGFBP-3 levels have strong positive correlations with the risk of DM in women [17]. The positive relationship between IGFBP-3 and lipid profiles in glucose intolerance state shown in the current study is consistent with the findings of reports about their metabolism [44, 45]. These findings suggest that measurement of total serum IGFBP-3 levels may be informative for determining its association with DM. However, the functional significance of IGFBP-3 in the pathoetiology of DM is largely unknown. Previous studies provided evidence for the direct involvement of IGFBP-3 in metabolic disorders, as increased IGFBP-3 fragments in circulation have been observed in catabolic states such as diabetes and obesity, leading to a decrease in total IGFBP-3 concentration [10, 46]. IGFBP-3 was also reported to inhibit cytokine-induced insulin resistance and early manifestation of atherosclerosis independent of IGF in the study by Mohanraj et al. [46]. They proved the fundamental concept regarding the proteolyzed IGFBP-3. The total IGFBP-3 decreased while proteolyzed IGFBP-3 increased in the serum of overweight and obese adolescents when compared with the control group. They reported that the sera of overweight and obese adolescents had significantly decreased intact IGFBP-3 through an increase in the production of proteolytic fragments. Consequently, the decreased circulating intact IGFBP-3 levels suppressed the anti-inflammatory and insulin-sensitizing functions of IGBPB-3 [46]. Interestingly, another observational study showed that total IGF-I and IGFBP-3 levels were not associated with the risk of DM [47].

Nevertheless, the current study demonstrated that serum IGF-I and total IGFBP-3 levels were not different among normal BMI, overweight, and obese subgroups and therefore were not correlated with BMI. This suggests that obesity has little impact on total IGFBP-3 levels in our study subjects.

From 2016 to 2018, in South Koreans aged 10 to 18 years, the prevalence of diabetes was 0.43% [48], and ours is one of the countries with the lowest incidence of diabetes. However, the prevalence of diabetes in our study population is high, and similar finding has already been reported by other authors. We had reported the prevalence of diabetes (36.4%, *n* = 40/110) in renal glycosuria in our province [18]. Moreover, of 21 students with glycosuria, 8 were diagnosed with diabetes [19], and in a Japanese study [20], of 298 glycosuric children, 133 (44.6%) students developed diabetes.

This study had some limitations. First, the comparison between NGT and IGT or DM groups was difficult due to the relatively small number of patients. Second, as we did not measure serum insulin levels during OGTT, we could not examine the relationships between IGF-I, IGFBP-3, and insulinogenic and disposition indexes. Third, our data did not include clinical parameters reflecting metabolic dysfunction adiposity, such as waist circumference (WC) and WC-to-height ratio, other than BMI. Finally, another limitation is that we did not measure serum-free IGF-I, IGFBP-1, and functional IGFBP-3 levels or perform genetic studies. Further studies are required to document the role of the IGF-I-IGFBP-3 axis in the development of glucose intolerance, especially in patients with T2DM and insulin resistance.

## 5. Conclusions

In summary, the present study showed that serum IGF-I and total IGFBP-3 levels are elevated in IGT and DM subjects with positive correlations with FPG and HbA1c; serum IGFBP-3 levels are also positively correlated with cholesterol levels. These findings suggest that the IGF-I-IGFBP-3 axis might be associated with diabetes risk and metabolic control in patients with glucose intolerance. Thus, we believe that the IGF-I-IGFBP-3 axis, especially IGFBP-3, could serve as a useful therapeutic target for the treatment of diabetes.

## Figures and Tables

**Table 1:** Demographic features of the study participants.

Characteristic	Total	NGT	Glucose intolerance			*P* value (NGT versus IGT versus DM)
			IGT	DM	Subtotal	
Number (%)	187 (100.0)	115 (61.5)	33 (17.6)	39 (20.9)	72	

Age (years)	12.51 ± 3.45	11.76 ± 3.56^*∗*^	12.83 ± 3.04^*∗*^	14.47 ± 2.54^†^	13.72 ± 2.88	<0.001

Sex (%)						
Female	98 (52.4)	51 (44.3)	20 (60.6)	27 (69.2)	47 (65.3)	0.004
Male	89 (47.6)	64 (55.7)	13 (39.4)	12 (30.8)	25 (34.7)	

Family history of DM in the first-degree relatives	63 (33.7)	24 (20.9)	16 (48.5)	23 (59.0)	39 (54.2)	<0.001
BMI (kg/m^2^)	24.50 ± 5.12	23.95 ± 4.68	25.20 ± 6.19	25.54 ± 5.29	25.39 ± 5.68	0.170
Normal	59 (31.6)	36 (31.3)	8 (24.2)	15 (38.5)	23 (31.9)	0.998
Overweight	42 (22.5)	30 (26.1)	5 (15.2)	7 (17.9)	12 (16.7)	
Obesity	86 (46.0)	49 (42.6)	20 (60.6)	17 (43.6)	37 (51.4)	

The data are presented as mean ± SD values. NGT, normal glucose tolerance; IGT, impaired glucose tolerance; DM, type 2 diabetes mellitus; BMI, body mass index; SD, standard deviation.

**Table 2:** Clinical characteristics of the study subjects.

Characteristic		Total	NGT	Glucose intolerance			*P* value (NGT versus IGT versus DM)
				IGT	DM	Subtotal	
Number (%)		187 (100.0)	115 (61.5)	33 (17.6)	39 (20.9)	72	
HbA1c (%)		6.33 ± 1.83	5.51 ± 0.29^*∗*^	6.07 ± 1.06†	9.00 ± 2.41‡	7.66 ± 2.40	<0.001
Systolic BP (mmHg)		120.24 ± 16.07	117.38 ± 15.23^*∗*^	123.34 ± 17.42^*∗*^†	125.68 ± 15.72†	124.61 ± 16.44	0.011
Diastolic BP (mmHg)		71.77 ± 11.31	71.27 ± 10.90	70.75 ± 12.38	74.05 ± 11.50	72.54 ± 11.94	0.367
FPG (mg/dL)		111.01 ± 53.26	89.04 ± 7.58^*∗*^	96.24 ± 15.30^*∗*^	188.28 ± 75.74†	146.10 ± 72.86	<0.001
2 hr PLG (mg/dL)		177.72 ± 128.10	115.34 ± 14.47^*∗*^	165.26 ± 30.96†	349.29 ± 110.88‡	277.35 ± 162.13	<0.001
C-peptide (ng/mL)		2.69 ± 1.54	2.34 ± 1.18^*∗*^	2.78 ± 1.21^*∗*^	3.56 ± 2.20†	3.21 ± 1.85	<0.001
Insulin (*μ*U/mL)		18.07 ± 17.66	15.21 ± 11.87^*∗*^	19.61 ± 13.15^*∗*^	26.67 ± 31.40†	23.14 ± 24.12	0.008
HOMA-IR		4.98 ± 5.78	3.34 ± 2.82^*∗*^	4.74 ± 3.40^*∗*^†	10.98 ± 10.09†	7.86 ± 8.10	<0.001

Lipid profile	Cholesterol (mg/dL)	169.24 ± 32.31	164.63 ± 30.27^*∗*^	165.06 ± 26.12^*∗*^	186.13 ± 37.53†	176.36 ± 34.65	0.001
	Triglyceride (mg/dL)	122.25 ± 69.34	112.50 ± 65.99^*∗*^	124.91 ± 67.74^*∗*^†	148.28 ± 74.81†	137.57 ± 72.12	0.020
	HDL (mg/dL)	46.55 ± 11.03	47.86 ± 11.45	45.09 ± 10.79	43.97 ± 9.52	44.49 ± 10.06	0.116
	LDL (mg/dL)	101.79 ± 30.75	97.96 ± 29.50^*∗*^	101.21 ± 27.52^*∗*^†	113.38 ± 34.56†	107.81 ± 31.90	0.025
IGF-I (ng/mL)		343.92 ± 153.32	303.70 ± 134.59^*∗*^	394.10 ± 140.16†	414.39 ± 178.38†	404.84 ± 160.67	<0.001
IGFBP-3 (ng/mL)		5688.01 ± 1229.54	5176.10 ± 953.61^*∗*^	6018.06 ± 1238.95†	6858.29 ± 1026.79‡	6463.64 ± 1199.37	<0.001
IGF-I/IGFBP-3 ratio		0.0599 ± 0.0221	0.0581 ± 0.0211	0.0654 ± 0.0226	0.0600 ± 0.0242	0.0625 ± 0.0234	0.276

The data are presented as mean ± SD values. NGT, normal glucose tolerance; IGT, impaired glucose tolerance; DM, type 2 diabetes mellitus; BP, blood pressure; HbA1c, glycosylated hemoglobin; FPG, fasting plasma glucose; 2 hr PLG, 2-hour postload glucose; HOMA-IR, homeostasis model assessment of insulin resistance; LDL, low-density lipoprotein; HDL, high-density lipoprotein; IGF-I, insulin-like growth factor-I; IGFBP-3, insulin-like growth factor binding protein-3; SD, standard deviation. ^*∗*^^, †, ‡^The same superscript designator indicates that there is no significant difference between the groups based on the Tukey multiple comparison test.

**Table 3:** Clinical characteristics of the study subjects with glucose intolerance according to BMI.

Characteristic		Total	Normal	Overweight	Obesity	*P* value
Number (%)		72 (100.0)	23 (31.9)	12 (16.7)	37 (51.4)	
Sex (male/female)		25/47	8/15	5/7	12/25	0.812
Age (yr)		13.72 ± 2.88	15.32 ± 2.47^*∗*^	13.13 ± 3.08†	12.92 ± 2.71†	0.004
BMI (kg/m^2^)		25.39 ± 5.68	20.41 ± 2.91^*∗*^	23.05 ± 5.03^*∗*^	29.24 ± 4.22†	<0.001
HbA1c (%)		7.66 ± 2.40	7.98 ± 2.92	7.55 ± 2.37	7.49 ± 2.10	0.743
Systolic BP (mmHg)		124.61 ± 16.44	117.48 ± 14.31^*∗*^	132.55 ± 15.59†	126.75 ± 16.63^*∗*^†	0.021
Diastolic BP (mmHg)		72.54 ± 11.94	70.87 ± 11.89	70.27 ± 7.43	74.31 ± 13.02	0.448
FPG (mg/dL)		146.10 ± 72.86	156.52 ± 75.47	150.08 ± 67.18WSY	138.32 ± 73.98	0.635
2 hr PLG (mg/dL)		277.35 ± 162.13	309.35 ± 142.62	319.08 ± 269.74	243.92 ± 121.64	0.197
C-peptide (ng/mL)		3.21 ± 1.85	1.87 ± 0.82^*∗*^	3.85 ± 2.32†	3.85 ± 1.73†	<0.001
Insulin (*μ*U/mL)		23.14 ± 24.12	9.04 ± 5.08^*∗*^	30.16 ± 27.33†	29.06 ± 26.77†	0.013
HOMA-IR		7.86 ± 8.10	4.08 ± 3.45^*∗*^	9.02 ± 7.07^*∗*^†	9.63 ± 9.52†	0.067
Lipid profile	Cholesterol (mg/dL)	176.47 ± 34.25	169.87 ± 40.92	183.33 ± 30.46	178.35 ± 30.98	0.491
	Triglyceride (mg/dL)	137.57 ± 72.12	95.09 ± 44.26^*∗*^	147.50 ± 68.86†	160.76 ± 76.64†	0.002
	HDL (mg/dL)	44.49 ± 10.06	48.74 ± 13.63^*∗*^	45.42 ± 8.11^*∗*^†	41.54 ± 6.74†	0.023
	LDL (mg/dL)	107.81 ± 31.90	100.26 ± 41.53	111.67 ± 21.12	111.24 ± 27.63	0.394
IGF-I (ng/mL)		404.84 ± 160.67	409.49 ± 123.89	441.71 ± 157.09	391.76 ± 182.44	0.682
IGFBP-3 (ng/mL)		6463.64 ± 1199.37	6390.00 ± 1079.20	6206.00 ± 1487.64	6587.06 ± 1203.85	0.643
IGF-I/IGFBP-3 ratio		0.0625 ± 0.0234	0.0646 ± 0.0187	0.712 ± 0.0233	0.0587 ± 0.0259	0.298

The data are presented as mean ± SD values. BMI, body mass index; BP, blood pressure; HbA1c, glycosylated hemoglobin; FPG, fasting plasma glucose; 2 hr PLG, 2-hour postload glucose; HOMA-IR, homeostasis model assessment of insulin resistance; LDL, low-density lipoprotein; HDL, high-density lipoprotein; IGF-I, insulin-like growth factor-I; IGFBP-3, insulin-like growth factor binding protein-3; SD, standard deviation. ^*∗*^^, †, ‡^The same superscript indicates that there is no significant difference between the groups based on the Tukey multiple comparison test.

**Table 4:** Simple correlation (*γ*) analysis between serum IGF-I and clinical variables.

		NGT (*n* = 115)		IGT (*n* = 33)		DM (*n* = 39)	
		*P*	*γ*	*P*	*γ*	*P*	*γ*
IGF-I	Age	<0.001	0.475	0.612	0.093	0.353	−0.159
	BMI	0.895	−0.013	0.487	0.127	0.080	−0.296
	FPG	0.014	0.242	0.482	−0.129	0.001	0.544
	2 hr PLG	0.965	0.004	0.252	−0.208	0.729	0.060
	Cholesterol	0.459	−0.074	0.514	0.120	0.820	−0.039
	Triglyceride	0.914	0.011	0.214	0.226	0.354	−0.159
	HDL	0.454	0.075	0.876	0.029	0.338	0.164
	LDL	0.148	−0.144	0.862	−0.032	0.897	−0.022
	HOMA-IR	0.004	0.294	0.349	0.184	0.176	0.268
	HbA1c	0.039	0.204	0.717	−0.067	0.028	0.367
	C-peptide	0.001	0.330	0.142	0.270	0.609	−0.088
	Insulin	0.008	0.270	0.279	0.212	0.914	−0.022
	IGFBP-3	<0.001	0.547	0.085	0.315	0.029	0.368

NGT, normal glucose tolerance; IGT, impaired glucose tolerance; DM, type 2 diabetes. BMI, body mass index; HbA1c, glycosylated hemoglobin; FPG, fasting plasma glucose; 2 hr PLG, 2-hour postload glucose; HOMA-IR, homeostasis model assessment of insulin resistance; LDL, low-density lipoprotein; HDL, high-density lipoprotein; IGF-I, insulin-like growth factor-I; IGFBP-3, insulin-like growth factor binding protein-3.

**Table 5:** Simple correlation (*γ*) between serum IGFBP-3 and clinical variables.

		NGT (*n* = 115)		IGT (*n* = 33)		DM (*n* = 39)	
		*P*	*γ*	*P*	*γ*	*P*	*γ*
IGFBP-3	Age	0.002	0.307	0.973	0.006	0.625	0.086
	BMI	0.031	0.215	0.006	0.480	0.967	−0.007
	FPG	0.117	0.158	0.304	0.191	0.012	0.421
	2 hr PLG	0.682	0.041	0.225	0.224	0.086	−0.294
	Cholesterol	0.547	0.061	0.029	0.392	0.008	0.442
	Triglyceride	0.008	0.266	0.013	0.441	0.200	0.222
	HDL	0.997	0.000	0.145	−0.268	0.529	0.110
	LDL	0.785	−0.028	0.018	0.423	0.047	0.354
	HOMA-IR	0.019	0.242	<0.001	0.670	0.294	−0.210
	HbA1c	0.003	0.296	0.019	0.420	0.004	0.477
	C-peptide	0.010	0.259	<0.001	0.661	0.068	−0.312
	Insulin	0.021	0.237	<0.001	0.626	0.070	−0.354
	IGF-I	<0.001	0.547	0.085	0.315	0.029	0.368

NGT, normal glucose tolerance; IGT, impaired glucose tolerance; DM, type 2 diabetes. BMI, body mass index; HbA1c, glycosylated hemoglobin; FPG, fasting plasma glucose; HOMA-IR, homeostasis model assessment of insulin resistance; LDL, low-density lipoprotein; HDL, high-density lipoprotein; IGF-I, insulin-like growth factor-I; IGFBP-3, insulin-like growth factor binding protein-3.

## Data Availability

The data used to support the findings of this study are available from the corresponding author upon reasonable request.
